# Trends and social inequalities in self-reported health and activity limitations in France between 2017 and 2021: results from four nationwide representative surveys

**DOI:** 10.1186/s12889-024-19437-2

**Published:** 2024-07-17

**Authors:** Hana Lahbib, Laure Carcaillon-Bentata, Nathalie Beltzer, Cyrille Delpierre, Joël Coste

**Affiliations:** 1 Santé Publique France (French National Public Health Agency), Direction Des Maladies Non Transmissibles, 12 Rue du Val d’Osne, Saint-Maurice, 94410 France; 2 CERPOP, UMR 1295, Inserm Université Toulouse III, Toulouse, France

**Keywords:** Self-reported health, Global activity limitation indicator, Trends, Health inequalities

## Abstract

**Background:**

Like other countries in Europe and around the world, France was hit by the COVID-19 pandemic in 2020, although it had also experienced several social crises since 2017. This study assessed the evolution of self-reported health and activity limitations and explored the dynamics of their socio-territorial inequalities among the French population aged 18–75 years between 2017 and 2021.

**Methods:**

Self-reported health (SRH) and global activity limitation indicator (GALI) were assessed in the same way in the four last editions of the French Health Barometer surveys conducted in the general population in 2017, 2019, 2020, and 2021, with between 9,200 and 24,500 subjects interviewed depending on the year. The prevalence of good or very good SRH and GALI (any limitation) and their evolution between 2017 and 2021 were studied according to sex, age, main socioeconomic positions (SEP), and regions. Poisson regression models were used to estimate adjusted prevalence ratios and potential modification effects of sociodemographic and geographic characteristics.

**Results:**

Between 2017 and 2021, SRH and GALI deteriorated in adults in France in a continuous way. Very good or good SRH decreased from 75.2% (CI_95%_ [74.5–75.9]) of subjects in 2017 to 68.5% (CI_95%_ [67.7–69.3]) in 2021. In parallel, GALI increased from 21.5% (CI_95%_ [21.0–22.2) in 2017 to 25.2% (CI_95%_ [24.5–26.0]) in 2021.The deterioration of indicators affected both sexes, all age classes (except 65–75 years), especially younger age classes (18–24 and 25–34 years), all geographical regions, and all SEP variables, with groups with a higher SEP deteriorating more than others. Negative variations exceeding 20% (8–10 percentage points on the absolute scale of indicators) were observed in several population groups from 2019 onwards.

**Conclusion:**

The previously observed deterioration of the SRH and GALI continued in France between 2017 and 2021, with narrowing socio-territorial gradients of inequalities. The impact of successive social and health crises on the poor evolution of self-reported health and activity limitations warrants further investigation over time and across locations using complementary and possibly more detailed indicators.

**Supplementary Information:**

The online version contains supplementary material available at 10.1186/s12889-024-19437-2.

## Introduction

Monitoring perceived health and quality of life in the general population has been a growing subject of interest in the last decades, as both indicators have been found to closely relate to and predict various health outcomes in the population [1]. Monitoring the perceived health in the population is particularly relevant in the context of health and social crises, as perceived health may be affected like other health indicators during such crises, which cause stress to people and care systems and have an impact on population health [2].

Common measures used to monitor the perceived health of populations in Europe and elsewhere, are the self-reported health (SRH) and global activity limitation indicator (GALI) from the Minimum European Health Module (MEHM) [3], a parsimonious set of global questions developed to allow comparisons between populations [4]. The SRH is used to measure health globally with a single question [4], with poor SRH being associated with poor outcomes such as mortality, functional limitations, and cognitive decline [5–7]. The GALI is also a single-question instrument assessing disability through activity limitations [4, 8]. This measure aims to assess subjects who identify health-related restrictions in their daily activities, especially among older populations. Activity limitations are a strong predictor of mortality and healthcare costs [8] and are also used in the assessment of the “Healthy Life Years” (HLY), a European indicator measuring disability-free life expectancy [9].

After the 2008 global financial crisis and its aftermath, which had a noticeable impact on quality of life [2, 10, 11], France, like other countries in Europe and around the world, was hit by the COVID-19 pandemic in 2020, although it had also experienced repeated terrorist attacks and several social crises since 2017 (e.g., yellow vest protests and violence in 2018, national strikes and blockades of public services and transport against the pension reform in 2019), which impaired transport capacities and autonomy and generated anxiety and distress in the population, among other impacts [12, 13]. Following the study of Carcaillon-Bentata et al. [14], which evidenced deteriorating self-reported health in France between 2010 and 2017, especially among individuals aged 55–65 and workers with an average level of education, this new investigation aimed to assess the evolution of self-reported health and activity limitations among the French population aged 18 to 75 years between 2017 and 2021. It especially considered trends in terms of age, sex, socioeconomic position, and regions in relation to socio-territorial inequalities, which were shown to substantially increase in this country since the 2000s [10].

## Methods

### Data sources

This study uses data from four French Health Barometer surveys conducted in 2017, 2019, 2020, and 2021. Since 1992, these surveys have been used as an epidemiological surveillance tool to monitor the main behaviors, attitudes, and perceptions of the French general population regarding their health. Health barometers, which have been regularly conducted by Santé Publique France since 1992, are among the data sources most used to assess the state of health of the French population. These barometers are cross-sectional telephone surveys with a random sampling method to ensure the representativeness of community-dwelling adults at the regional level, with the random generation of landline and mobile phone numbers in an overlapping dual-frame approach [15]. To be included in the survey, individuals have to live in metropolitan France and speak French.

In 2017, the survey included people aged 18 to 75 years; in 2019, 2020, and 2021, the survey was extended to include those aged 18 to 85 years. Residents of institutions, collective housing, and hospitals were excluded. Regarding the 2020 Barometer, its investigation field took place during the COVID-19 pandemic and was disrupted due to the implementation of the first lockdown measures. Therefore, the investigation was prematurely ended before the desired sample size was achieved, although this did not affect its representativeness. The pre-lockdown sample size was nevertheless used to carry out analysis at the national level. Subjects were included from January 8 to March 16, 2020.

In 2020, the sample included 9,178 subjects, with a participation rate of 40.0% [16]. In 2017, the survey included 25,319 individuals, with a participation rate of 48.5% [17]. In 2019, 10,352 individuals were included, with a participation rate of 50.8% [18]. In 2021, 24,514 subjects were included, with a participation rate of 44.3% [19].

To be as representative as possible of the French population, the data of the four surveys were weighted by calibration in terms of age, sex, region, town size, education level, and number of people per household. For the 2017 Barometer, the population structure was provided by the 2016 Labor Force Survey of the French population [20]. Data from the 2019 and 2020 Barometers were calibrated to the structure of the 2018 Labor Force Survey [21]. Regarding the 2021 Barometer, the calibration of the sample was based on the 2020 Labor Force Survey [22]. The Labor Force Surveys are conducted by INSEE (Institut National de la Statistique et des Etudes Economiques, Paris).

The detailed methodology and questionnaires of the four barometers are available online [16–19, 23–26].

### Health indicators

In the four surveys, identical wording was used for the SRH and GALI questions [3], respectively:

- “How is your health in general? Is it…” with five possible answers: “Very good, good, fair, bad, very bad”;
- “For at least 6 months, to what extent have you been limited because of a health problem in the activities people usually do?” with three possible answers: “Severely limited, limited but not severely, not limited at all.”

Following WHO recommendations [27] and in line with EUROSTAT use [28], the SRH responses were classified into two groups: “very good, good” versus “fair, bad, very bad” (i.e., “less than good”) health. The GALI responses were also classified into two groups: “severely limited, limited but not severely” (i.e., “any limitation”) versus “not limited at all” in line with EUROSTAT use [29].

### Statistical analysis

As the age limit in the 2017 Barometer was 75 years, the analyses of this study focus on the population aged 18 to 75 years in all four surveys; subjects aged 76 to 85 years in the 2019, 2020, and 2021 Barometers were thus excluded. For the 2020 and 2021 Barometers, 8,473 and 22,625 subjects were included, respectively. In the 2019 Barometer, 9,460 individuals were aged between 18 and 75 years, although the MEHM questions were asked to only a subsample (roughly half of the sample, 4,909 individuals), representative of the population.

For each barometer, the population was described regarding sociodemographic and geographic variables: sex; age (six classes: 18–24, 25–34, 35–44, 45–54, 55–64, 65–75 years); socioeconomic position (SEP) measured according to socio-professional category (divided into three categories: farmer/manual worker, employee/middle manager, executive/intellectual profession), education level (< high school diploma, high school diploma, > high school diploma), and monthly income (four categories: < 1,500€, 1,500€-3,000€, > 3,000€, not reported); and geographic region (five main regions: North-East, North-West, South-East, South-West, and greater Paris region, based on groupings of the 13 administrative regions created in 2018). SRH (as five and two categories) and activity limitations (as three and two categories) were also described.

Values and absolute changes of “less than good SRH” and “any activity limitation” indicators were described for each Barometer year and for each of the sociodemographic and geographic variables considered here.

Poisson regression models were constructed to assess the relative risk of “less than good SRH” and “any activity limitation” between 2017 and 2021 (dependent variables) while encoding the Barometer years as dummy variables and using 2017 as the reference year in order to obtain prevalence ratios (PRs) and 95% confidence intervals (CI), adjusted for all other covariates or independent variables: age, sex, socio-professional category, education level, monthly income, and geographic region as described above [30]. Effect modifications of age, education level, socio-professional category, monthly income, and geographic region on the relationship between Barometer years and the two health outcomes were explored by examining interaction terms between Barometer years and all the modifiers within the fully adjusted model [31]. In the case of significant interaction terms (*p* < 0.05), stratified Poisson regression analyses of the risk of “less than good SRH” and “any activity limitation” according to Barometer year were subsequently performed, while adjusting for all covariates except for the modifier under consideration. Analyses regarding education level, socio-professional category, and income were run while excluding the category of 18–24 year-olds due to the high proportion of students in this age group for whom these variables are not relevant.

All analyses were performed using appropriate weights with the software SAS 9.4. Weights took into account the selection probability of the individual and were calibrated to adjust for the French population demographic structure of the year in question in terms of sex by age (10-year intervals), region, level of urbanization of the place of residence, household size, and education level [15].

## Results

The main (and very similar) characteristics of the population included in the four surveys are summarized in Supplementary Table 1. In 2017, 75.2% (CI_95%_ [74.5–75.9]) of the sample reported a very good or good SRH. This prevalence decreased thereafter, from 73.3% in 2019 to 68.3% in 2020 and 68.5% (CI_95%_ [67.7–69.3]) in 2021. In parallel, the proportion of subjects reporting (any) limitation in activities increased from 21.5% (CI_95%_ [21.0–22.2]) in 2017 and 23.1% in 2019 to 25.7% in 2020 and 25.2% (CI_95%_ [24.5–26.0]) in 2021.

### Factors associated with SRH and activity limitations, and effect modifiers

Sex, age, socio-professional category, education level, monthly income, geographic region, and Barometer year were independently associated with SRH and (any) limitation (Table 1 and Supplementary Table 2, excluding individuals under 25 years). “Dose–effect” gradients were observed for age and all SEP variables for both indicators: older age was associated with a higher proportion of less than good SRH and activity limitations, while a lower socioeconomic level was also associated with a higher proportion of less than good SRH and limitations. The North-East and Paris regions generally had the highest proportion of less than good SRH compared with the other regions, whereas the differences were not significant for limitations.

**Table 1:**
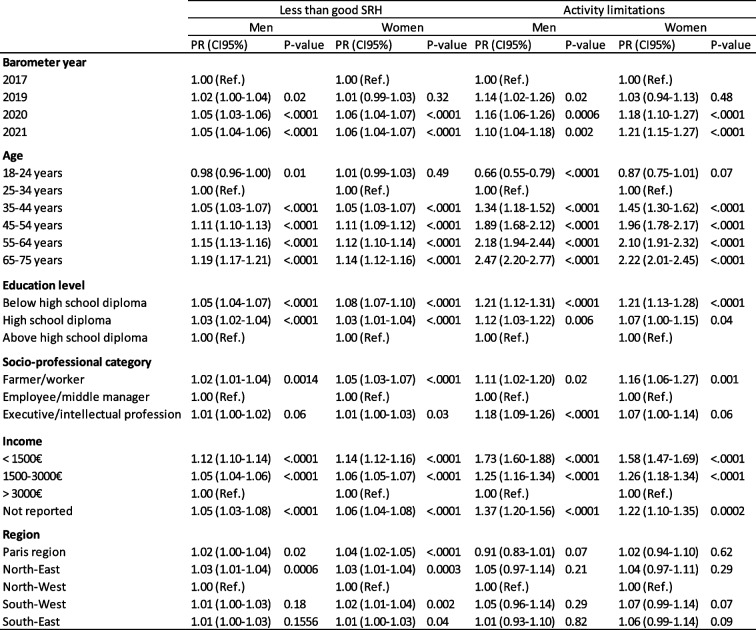
Final models of factors independently associated with less than good self-reported health and (any) limitation. Prevalence ratios of less than good self-reported health and activity limitations associated with barometer years and other covariates^a^

^a^Prevalence ratios (PR) and 95% confidence intervals (CI) estimated through Poisson regression models with less than good self-reported health or activity limitations as the dependent variable, and barometer years and covariates as the independent variables

From 2017 onwards, adjusted PRs of less than good SRH and (any) activity limitations associated with Barometer year increased, albeit non-significantly for both sexes. They reached a maximum in 2020 for all indicators except for activity limitations in women for whom the increase was the greatest in 2021.

Effect modification of age, education level, socio-professional category, and monthly income but not geographic region were frequently found for the relationship between Barometer years and the two health outcomes (Supplementary Table 3).

### Evolution of SRH and activity limitations according to age

**Fig. 1:**
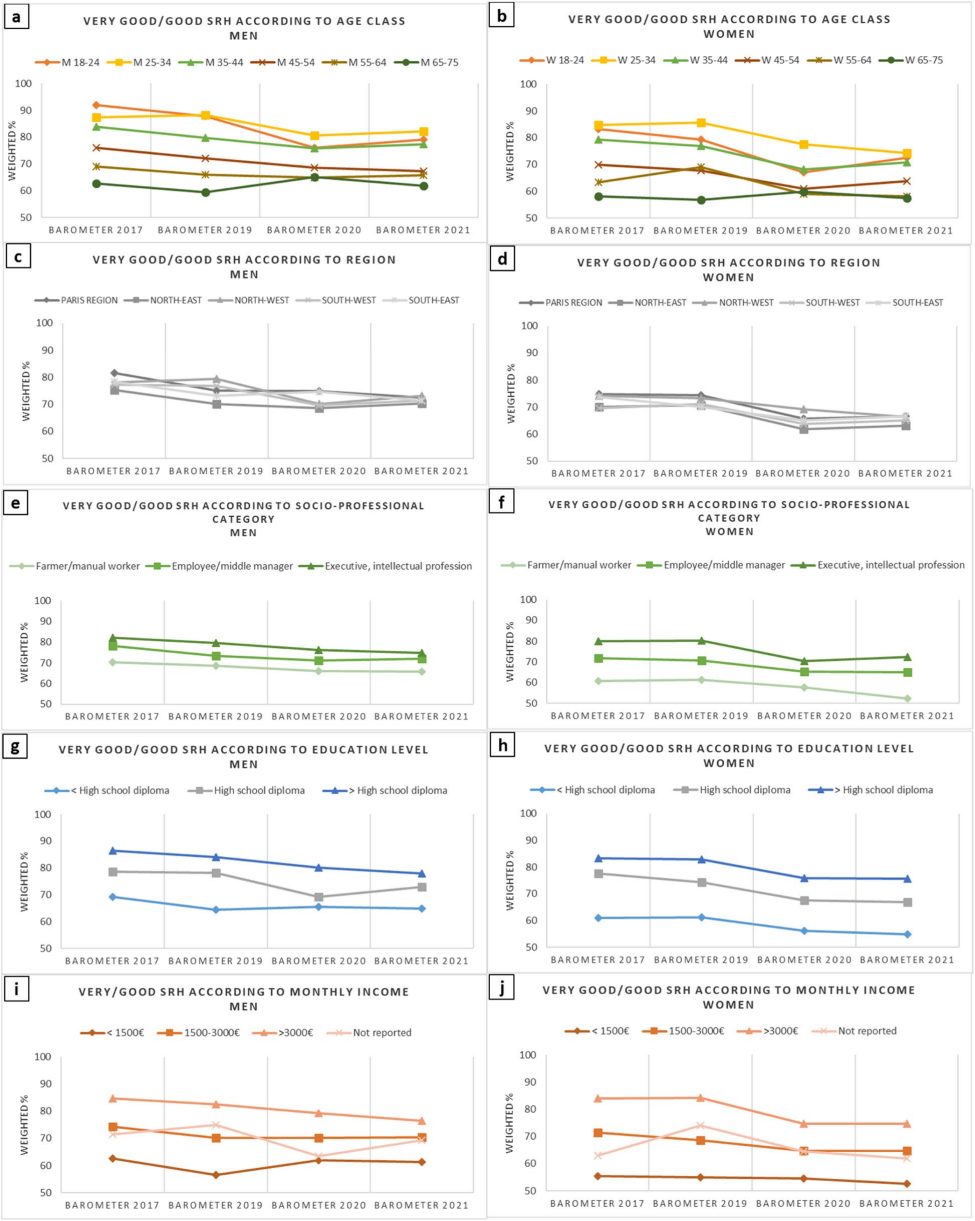
Evolution of prevalence of self-reported health (SRH) among men and women (Health Barometers from 2017, 2019, 2020, and 2021). Legend: Evolution of prevalence of self-reported health (SRH) among men and women according to age class, French region, socio-professional category*, education level*, and monthly income* (Health Barometers from 2017, 2019, 2020, and 2021). * Excluding 18–25 year-olds

The evolution of SRH according to age classes in men is presented in Fig. 1a. The prevalence of very good/good SRH decreased linearly in all age classes between 2017 and 2021, except older adults (65–75 years). In 2017, a clear-cut gradient of SRH with age was observed, with younger subjects having a better SRH. This gradient was reduced in 2021 with the grouping of several age classes, notably younger (SRH_18-24_ = 79.2%, SRH_25-34_ = 82.2% and SRH_35-44_ = 77.4%) and middle-aged subjects (SRH_45-54_ = 67.4%, SRH_55-64_ = 65.8%), which were closer to older subjects (SRH_65-75_ = 61.9%).

The deterioration of SRH was especially pronounced in young men (18–24 years), decreasing from 92.0% of positive SRH in 2017 to 79.2% in 2021 (decrease of 13 percentage points), with an even lower score in 2020 (76%).

Results showing the modeling of the prevalence of less than good SRH between 2017 and 2021 are presented in Table 2. In men, the deterioration was already large in 2020 in the two younger age classes, especially the youngest: PR_18-24_ = 1.14, CI_95%_ [1.09–1.20]; PR_25-34_ = 1.06, CI_95%_ [1.02–1.10]. The deterioration was more progressive in the two following age classes, where the greatest deterioration was observed in 2021: PR_35-44_ = 1.06, CI_95%_ [1.03–1.08]; OR_45-54_ = 1.08, CI_95%_ [1.05–1.11]. In the two oldest age classes (55–64 and 65–75 years), the deterioration was quite small and even non-significant in the latter class.

**Table 2:**
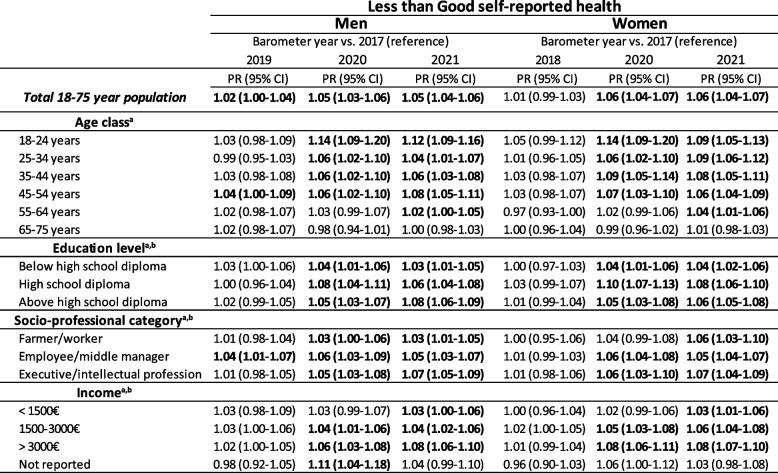
Evolution of less than good self-reported health prevalence ratios^a^ between 2017 and 2021 across age, education, socio-professional category, and income (Health Barometers from 2017, 2019, 2020, and 2021)

^a^Prevalence ratios (PR) and 95% confidence interval (CI) estimated through Poisson regression models with less than good self-reported health as the dependent variable, and barometer years and covariates as the independent variables (except for the stratified variable) in each presented strata (in the rows)

^b^Excluding 18-25 year-olds

The evolution of the prevalence of very good/good SRH according to age classes in women is presented in Fig. 1b. The deterioration of SRH was more marked in young women, with a decrease in the prevalence of very good/good SRH of 10 percentage points between 2017 and 2021 among 18–24 year-olds (from 83.2% to 72.5%, with the lowest point at 67% in 2020) and among 25–34 year-olds (from 84.8% to 74.2%). A decrease of 9 percentage points occurred among 35–44-year-old women (79.3% in 2017 and 70.8% in 2021, with the lowest point at 68.2% in 2020). The deterioration of SRH was marked (Table 2) among all age classes of women, except the oldest (65–75 years), with the temporal patterns and PRs being very similar to those found in men.

**Fig. 2:**
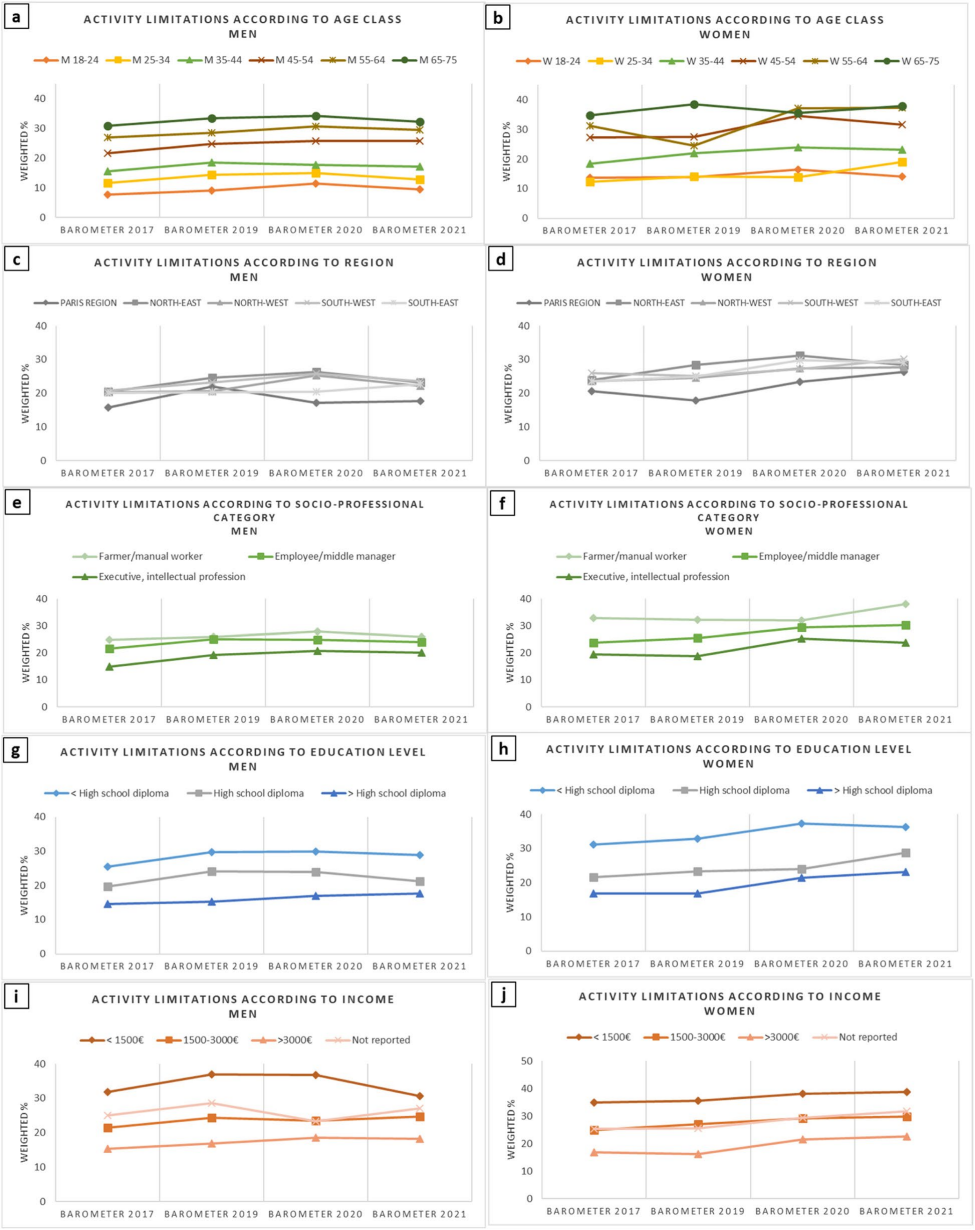
Evolution of prevalence of activity limitations among men and women (Health Barometers from 2017, 2019, 2020, and 2021). Legend: Evolution of prevalence of activity limitations among men and women according to age class, French region, socio-professional category*, education level*, and monthly income* (Health Barometers from 2017, 2019, 2020, and 2021). * Excluding 18–25 year-olds

The evolution of the prevalence of activity limitations between 2017 and 2021 according to age classes among men is presented in Fig. 2a. An increase was observed in all age classes but was only significant among 45–54-year-old men (PR_45-54_ = 1.23, CI_95%_ [1.07–1.41]) (Table 3). In women, the increase was generally more marked than in men and observed in all age classes, which was already evident in 2020 except in the youngest (18–24 years) and oldest classes (55–64 years) (Fig. 2b). It was especially high and significant in the following age classes: 25–34 years (PR_25-34_ = 1.52, CI_95%_ [1.26–1.84]) (in 2021), 35–44 years (PR_35-44_ = 1.32, CI_95%_ [1.07–1.62]), 45–54 years (PR_45-54_ = 1.24, CI_95%_ [1.07–1.43]), and 55–64 years old (PR_55-64_ = 1.20, CI_95%_ [1.06–1.36]) (in 2020) (Table 3).

**Table 3:**
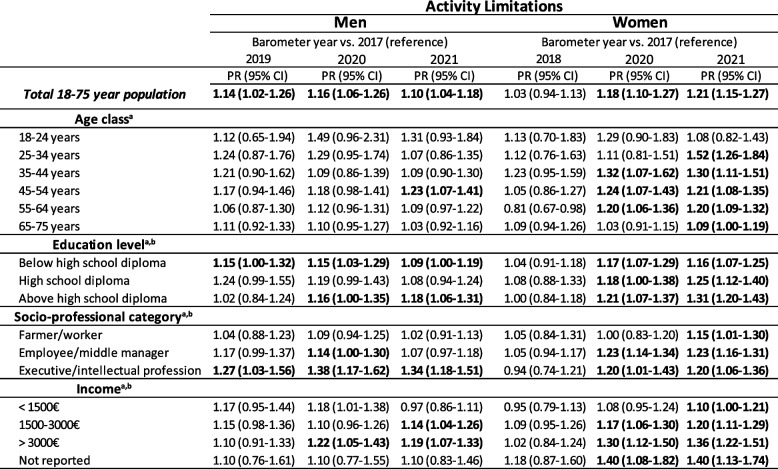
Evolution of activity limitations between 2017 and 2021 across age, education, socio-professional category, and income (Health Barometers from 2017, 2019, 2020, and 2021)

^a^Prevalence ratios (PR) and 95% confidence interval (CI) estimated through Poisson regression models with activity limitations as the dependent variable, and barometer years and covariates as the independent variables (except for the stratified variable) in each presented strata (in rows)

^b^Excluding 18-25 year-olds

### Evolution of SRH and activity limitations according to regions

Figures 1c, d, and 2c, and d respectively show the evolution of SRH and activity limitations between 2017 and 2021 across the different French geographic regions. The deterioration of both indicators was generalized across regions and sexes, and the gradients observed in 2017 tended to vanish in 2021 except for activity limitations in men (Fig. 2c). The deteriorations were similar in all regions, with no interaction being found between the Barometer year and region (Supplementary Table 2).

### Evolution of SRH and activity limitations according to socioeconomic position

Regarding SEP, all the socio-professional (Figs. 1e, f), education (Figs. 1g, h), and monthly income categories (Figs. 1i, j) were impacted by the decrease in the prevalence of very good/good SRH over time in both men and women. All the deteriorations were significant from 2020 onwards, with the higher socio-professional, education, and income categories being more impacted in both men and women (Table 2). The deterioration was particularly pronounced in highly educated (decrease in men from 86.5 in 2017 to 77.9 in 2021 and slightly less in women from 83.3 in 2017 to 75.6 in 2021) and high-income groups (decrease of 8 and 9 percentage points in men and women, respectively).

Regarding the prevalence of activity limitations according to SEP, its evolution differed slightly more according to sex. In women, all socio-professional, education, and monthly income categories were largely and significantly impacted, especially in the medium and higher categories for which the increases reached 6 to 7 percentage points and PRs ranged up to 1.30 or 1.40 (Figs. 2f, h, j, Table 3). In men, however, the results were more heterogeneous, as the prevalence of activity limitations increased in all socio-professional (Fig. 2e), education (Fig. 2g), and income categories (Fig. 2i), although the increases only reached 5 percentage points and PRs rarely exceeded 1.30, except for executive/intellectual professions (PR = 1.38, CI_95%_ [1.17–1.62]) in 2020 (Table 3).

## Discussion

Based on the last four nationwide representative surveys of the French Health Barometer, this study showed a continuous and linear deterioration in self-reported health and, symmetrically, activity limitations in adults in France between 2017 and 2021. This deterioration concerns a time period much longer than the COVID-19 crisis, preceding it to a large extent, and affects both sexes and all age classes (except 65–75 years), especially the younger groups (18–24 and 25–34 years). This deterioration led to a narrowing or even a disappearance of the regularly spaced age gradients usually observed with the SRH and GALI. A similar deterioration was observed for both sexes in all geographical regions, and for all studied SEP variables (socio-professional category, education, income), with the groups with higher SEP levels falling the most (up to 8 and 9 percentage points for some). To our knowledge, this is the first study to consider recent trends before and during the COVID-19 crisis in both SRH and activity limitations at a nationwide level in relation to sex, age, and socioeconomic and geographic indicators.

### Impact of the French social and health crises on self-reported health and limitations

Like several countries such as the UK, Italy, and Belgium, France imposed unprecedented and widespread lockdowns several times during the COVID-19 crisis, which caused stress and psychological distress to large parts of the population, especially younger people [32]. In France, however, the COVID-19 pandemic occurred after several successive social crises, including the terrorist attacks that have not ceased since 2012, the yellow vest protests and related violence in 2018 and the strikes against the pension reform in 2019 (eventually abandoned), which severely affected public transport, public services, and several sectors of the economy. In France, quality of life and perceived health have steadily fallen since the late 1990s [10, 33]. The present study showed the continuous deterioration of SRH and GALI in the younger age classes (18–34 years). By contrast, the trends concerning the older retired groups were less marked, thus suggesting that these groups were less impacted by the recent social and health crises than the active populations, which may endure job or income losses as a result of these crises. These results are in line with the last trend observed between 2010 and 2017, where a general decrease in SRH was observed in all age classes but was less significant as age increased [14].

The general and substantial decrease in SRH and more moderate increase in activity limitations observed in this study are consistent with the findings of several recently published studies concerning France, which showed the degradation of several heath indicators since 2017, that is, before the COVID-19 crisis and in line with the trends of previous years: i) an increased prevalence of major depressive disorders since 2010 with an acceleration between 2017 and 2021, especially among young adults [34]; ii) a large increase in the prescription of psychotropic drugs (anxiolytics, hypnotics, antidepressants) between 2018 and 2021 [35]; and iii) a new increase in tobacco smoking from 2019 onwards, notably in women and less-educated individuals after a stabilization period of several years [36]. This deterioration of mental health indicators was already reported in the aftermath of negative socioeconomic changes (job loss, household income reduction) in the Netherlands at the end of the 2000s [37]. Detailed long-term studies of SRH and activity limitations that precede and include the recent crises (both economic and social) are still lacking in European countries, although our results are consistent with the overall trends observed for the 2013–2021 period in Eurostat SILC statistics for several European countries, notably in working age categories [38, 39]. Our results are also consistent with recent trends in healthy life expectancies reported in several European countries [40]. However, further research is needed to precisely quantify the various health impacts of economic, social, and political crises, whether these impacts are objective, such as job loss or income reduction, or more subjective, such as a feeling of decline, lower trust in institutions, and reduced expectations.

### Impact on health inequalities

Despite the observation that the COVID-19 pandemic and associated crisis differentially impacted populations according to sex, age, SEP, and territorial location and thus increased health inequalities in terms of morbidity and mortality [41, 42], the assessment of the differential impact of the crisis on the perceived health and quality of life of (surviving) populations is much less straightforward. Differentiated and sometimes contradictory variations in these measures have been reported from the first weeks of the pandemic [43–45], suggesting that the impact of the crisis on perceived health and quality of life may have been different depending on the SEP, the timing of the studies with respect to the COVID-19 pandemic, as well as the country and its pre-crisis level of perceived health and dynamics [46].

Our results may seem paradoxical, as they show the narrowing (rather than the growth) of the gradients of inequalities of SRH and activity limitations across ages and social indicators, as well as the greater negative impact on the healthier population groups i.e., the younger male and wealthier populations. However, a floor effect may have occurred with these indicators, which would have prevented those in the lowest categories, which had already fallen in the early 2000s [33], from declining further. Moreover, since perceived health and quality-of-life measures have been shown to be influenced by people’s expectations [47, 48] and life satisfaction [49], which are dependent on the socioeconomic context and social crises, different levels of satisfaction and expectations may have contributed to these results: as the formerly more privileged groups may have had greater expectations, they would have been more affected in the event of crises such as those occurring between 2017 and 2021 [50, 51]. The major role played by expectations may also explain why the gradient narrowing was more marked with the SRH than with the GALI, as the latter indicator is probably less “subjective.” Similar and varied trends with the narrowing or enlargement of gradients depending on the indicators were recently reported for older populations in European countries [52], although the explanations were not obvious, as in our own study (note that a floor effect may have also influenced expectations in the less privileged populations).

### Strengths and limitations

This study has a number of strengths, including the national representativeness of the survey samples, the interviews conducted using the same methodology and the same set of validated questions, the large number of participants, and especially the number of time points (*n* = 4), which allowed the investigation of nonlinear trends. The study also has several limitations, including the dichotomization of indicators and the resulting loss of information, the time frame (four surveys in 5 years), which is relatively short in view of the temporal changes that probably began beforehand, although it included three evaluation points before the COVID-19 crisis. Moreover, the sample size might have limited the power of some of the stratified analyses, while the correction for multiple testing was not performed, as this is not routinely conducted in survey analyses.

## Conclusion

This study reports the continued negative trends of SRH and activity limitations in France between 2017 and 2021 in a context marked by successive health and social crises. It also evidenced differences in trends according to sex, age, SEP, and geographical region, with the formerly privileged groups being most affected by the negative trends with a reduction in socio-territorial and age gradients. Whereas a definitive interpretation of these results may take time (i.e., before trends across more studies can be observed), they nonetheless advocate for the collection of more data over time and across locations using complementary and possibly more detailed measures (including multidimensional quality-of-life instruments). They also call for the analysis of these data as precisely as possible, while taking into account the social and psychological context of the data collection.

### Supplementary Information

Supplementary Material 1: Supplementary Table 1. Characteristics of the four barometer samples (Health Barometers from 2017, 2019, 2020, and 2021). Supplementary Table 2. Final models of factors independently associated with less than good self-reported health and (any) limitation, excluding 18-24 year-olds. Prevalence ratios of less than good self-reported health and activity limitations associated with barometer years and other covariates. Supplementary Table 3. Effect modification of age, education level, socio-professional category, monthly income, and geographic region on the relationship between Barometer years. *P*-values of the interaction terms between Barometer years and the potential modifier within the fully adjusted model are reported.

## Data Availability

The datasets used and analyzed during the current study are not publicly available. Data are available on request from Santé publique France. Any request must be addressed to Jean-Baptiste Richard (Jean-Baptiste.RICHARD@santepubliquefrance.fr).

## Abbreviations

SRH: Self-reported health
GALI: Global activity limitations indicator
WHO: World health organization
SEP: Socioeconomic positions
OR: Odds-ratio
