# Supplementary material for: Trends and social inequalities in self-reported health and activity limitations in France between 2017 and 2021: results from four nationwide representative surveys
Source: BMC Public Health. 2024 Jul 17;24:1916. doi: 10.1186/s12889-024-19437-2 (PMC11253369; doi:10.1186/s12889-024-19437-2)
Supplement: Supplementary file 1 — Supplementary Material 1: Supplementary Table 1. Characteristics of the four barometer samples (Health Barometers from 2017, 2019, 2020, and 2021). Supplementary Table 2. Final models of factors independently associated with less than good self-reported health and (any) limitation, excluding 18-24 year-olds. Prevalence ratios of less than good self-reported health and activity limitations associated with barometer years and other covariates. Supplementary Table 3. Effect modification of age, education level, socio-professional category, monthly income, and geographic region on the relationship between Barometer years. P-values of the interaction terms between Barometer years and the potential modifier within the fully adjusted model are reported. [file 12889_2024_19437_MOESM1_ESM.docx]

Supplementary Table 1: Characteristics of the four barometer samples (Health Barometers from 2017, 2019, 2020, and 2021).

|  | **2017 Barometer** | | **2019 Barometer** | | **2020 Barometer** | | **2021 Barometer** | |
| --- | --- | --- | --- | --- | --- | --- | --- | --- |
|  | n = 25,319 | | n = 4,909 | | n = 8,473 | | n = 22,625 | |
|  | **n (%)** | **weighted %** | **n (%)** | **weighted %** | **n (%)** | **weighted %** | **n (%)** | **weighted %** |
| **Sex** |  |  |  |  |  |  |  |  |
| Men | 11,596 (45.8) | 48.7 | 2,240 (45.6) | 48.7 | 3,932 (46.4) | 48.6 | 10,629 (47) | 48.6 |
| Women | 13,723 (54.2) | 51.3 | 2,669 (54.4) | 51.3 | 4,541 (53.6) | 51.4 | 11,996 (53) | 51.4 |
| **Age (years)** |  |  |  |  |  |  |  |  |
| 18-24 | 2,277 (9) | 11.2 | 377 (7.7) | 11.3 | 708 (8.4) | 11.3 | 2,035 (9) | 11.5 |
| 25-34 | 3,717 (14.7) | 17.1 | 656 (13.4) | 16.6 | 1,065 (12.6) | 16.6 | 3,220 (14.2) | 16.2 |
| 35-44 | 4,397 (17.4) | 18.5 | 803 (16.4) | 17.8 | 1,370 (16.2) | 17.8 | 3,851 (17.0) | 17.7 |
| 45-54 | 5,010 (19.8) | 19.5 | 1,007 (20.5) | 19.3 | 1,680 (19.8) | 19.3 | 4,530 (20.0) | 18.8 |
| 55-64 | 5,264 (20.8) | 18.2 | 1,067 (21.7) | 18,0 | 1,839 (21.7) | 18 | 4,662 (20.6) | 18.2 |
| 65-75 | 4,654 (18.4) | 15.5 | 999 (20.4) | 17.1 | 1,811 (21.4) | 17 | 4,327 (19.1) | 17.6 |
| **Socio-professional category** | *MD = 262* |  | *MD = 42* |  | *MD = 74* |  | *MD = 347* |  |
| Farmer/manual worker | 5,025 (20.1) | 25.5 | 975 (20) | 25.9 | 1,586 (18.9) | 25.3 | 3,870 (17.4) | 23.6 |
| Employee/middle manager | 13,934 (55.6) | 53.6 | 2,691 (55.3) | 52.7 | 4,595 (54.7) | 52.3 | 11,895 (53.4) | 51.9 |
| Executive/intellectual profession | 6,098 (24.3) | 20.9 | 1,201 (24.7) | 21.5 | 2,218 (26.4) | 22.4 | 6,513 (29.2) | 24.5 |
| **Monthly income** |  |  |  |  |  |  |  |  |
| < 1500€ | 4,572 (19) | 22.5 | 791 (17.9) | 21.5 | 1,657 (21.3) | 26.2 | 3,966 (19) | 24.8 |
| 1500-3000€ | 10,225 (42.4) | 42.5 | 1,886 (42.7) | 43.6 | 3,374 (43.4) | 43.3 | 8,792 (42.1) | 41.9 |
| >3000€ | 9,334 (38.7) | 35.0 | 1,743 (39.4) | 34.9 | 2,737 (35.2) | 30.5 | 8,139 (39) | 33.3 |
| Not reported | 1,188 (4.7) | 5.5 | 489 (10) | 10.9 | 705 (8.3) | 9.7 | 1,728 (7.6) | 8.9 |
| **Education level** | *MD = 61* |  | *MD = 18* |  | *MD = 35* |  | *MD = 93* |  |
| < High school diploma | 9,609 (38.0) | 48.7 | 1,785 (36.5) | 47.2 | 2,920 (34.6) | 47.3 | 6,774 (30.1) | 44.2 |
| High school diploma | 5,422 (21.5) | 20.4 | 1,073 (21.9) | 20.2 | 1,793 (21.3) | 20.3 | 4,768 (21.2) | 20.8 |
| > High school diploma | 10,227 (40.5) | 30.9 | 2,033 (41.6) | 32.6 | 3,725 (44.2) | 32.4 | 10,990 (48.8) | 35.1 |

Supplementary Table 1 (continued): Characteristics of the four barometer samples (Health Barometers from 2017, 2019, 2020, and 2021).

|  | **2017 Barometer** | | **2019 Barometer** | | **2020 Barometer** | | **2021 Barometer** | |
| --- | --- | --- | --- | --- | --- | --- | --- | --- |
|  | n = 25,319 | | n = 4,909 | | n = 8,473 | | n = 22,625 | |
|  | **n (%)** | **weighted %** | **n (%)** | **weighted %** | **n (%)** | **weighted %** | **n (%)** | **weighted %** |
| **Self-perceived health** | *MD = 27* |  | *MD = 10* |  | *MD = 9* |  | *MD = 39* |  |
| Very good | 7,989 (31.6) | 31.6 | 1,566 (32.0) | 32.0 | 2,182 (25.8) | 26.0 | 5,885 (26.1) | 26.1 |
| Good | 1,1252 (44.5) | 43.6 | 2,100 (42.9) | 41.3 | 3,738 (44.2) | 42.2 | 9,942 (44.0) | 42.4 |
| Fair | 4,690 (18.5) | 18.8 | 959 (19.6) | 20.3 | 2,059 (24.3) | 25.0 | 5,369 (23.8) | 24.5 |
| Bad | 1,120 (4.4) | 4.8 | 222 (4.5) | 5.4 | 414 (4.9) | 5.5 | 1,198 (5.3) | 5.9 |
| Very bad | 241 (0.95) | 1.2 | 52 (1.1) | 1.2 | 71 (0.8) | 1.3 | 192 (0.9) | 1.1 |
| **Self-perceived health (two classes)** |  |  |  |  |  |  |  |  |
| Very good or good | 19,241 (76.1) | 75.2 | 3,666 (74.8) | 73.3 | 5,920 (69.9) | 68.3 | 15,827 (70.1) | 68.5 |
| Less than good | 6,051 (23.9) | 24.8 | 1,233 (25.2) | 26.7 | 2,544 (30.1) | 31.8 | 6,759 (29.9) | 31.5 |
| **Activity limitations** | *MD = 28* |  | *MD = 9* |  | *MD = 12* |  | *MD = 46* |  |
| Severely limited | 1,534 (6.1) | 6.6 | 347 (7.1) | 7.4 | 716 (8.5) | 9.4 | 1,962 (8.7) | 9.3 |
| Limited but not severly | 3,913 (15.5) | 15.0 | 786 (16.0) | 15.8 | 1,462 (17.3) | 16.2 | 3,702 (16.4) | 15.9 |
| Not limited at all | 19,844 (78.5) | 78.4 | 3,767 (76.9) | 76.8 | 6,283 (74.3) | 74.4 | 16,915 (74.9) | 74.8 |
| **Activity limitations (two classes)** |  |  |  |  |  |  |  |  |
| No | 19,844 (78.5) | 78.4 | 3,767 (76.9) | 76.8 | 6,283 (74.3) | 74.4 | 16,915 (74.9) | 74.8 |
| Yes | 5,447 (21.5) | 21.6 | 1,133 (23.1) | 23.3 | 2,178 (25.7) | 25.6 | 5,664 (25.1) | 25.2 |
| *MD = Missing Data* |  |  |  |  |  |  |  |  |

Supplementary Table 2: Final models of the factors independently associated with less than good self-reported health and (any) limitation, excluding 18-24 year-olds. Prevalence ratios of less than good self-reported health and activity limitations associated with barometer years and other covariates*.

* Prevalence ratios (PR) and 95% confidence intervals (CI) estimated through Poisson regression models with less than good self-reported health or activity limitations as the dependent variable, and barometer years and covariates as the independent variables.

Supplementary Table 3: Effect modification of age, education level, socio-professional category, monthly income, and geographic region on the relationship between Barometer years. P-values of the interaction terms between Barometer years and the potential modifier within the fully adjusted model are reported.

|  | Self-Reported health | | Activity limitations | |
| --- | --- | --- | --- | --- |
| Interaction | Male | Female | Male | Female |
| Year and Age | **<0.0001** | **<0.0001** | 0.18 | **0.02** |
| Year and Education level* | **0.0004** | 0.09 | 0.34 | 0.04 |
| Year and Socio-professional category* | **0.02** | 0.90 | **0.002** | 0.37 |
| Year and Income* | **0.004** | **0.04** | 0.09 | **0.001** |
| Year and Region | 0.29 | 0.33 | 0.97 | 0.58 |

* Excluding 18-25 year-old
